# Grand Challenge Veterinary Neurology and Neurosurgery: Veterinary Neurology and Neurosurgery – Research for Animals and Translational Aspects

**DOI:** 10.3389/fvets.2015.00013

**Published:** 2015-05-26

**Authors:** Andrea Tipold

**Affiliations:** ^1^Department of Small Animal Medicine and Surgery, University of Veterinary Medicine Hannover, Hannover, Germany

**Keywords:** veterinary neurology, translational medicine, challenges, clinical research, animal species

Neurology and neurosurgery are a recognized specialty in human and veterinary medicine (1) having many overlaps with other disciplines, such as internal medicine, surgery, imaging, and pathology. In some countries, in human medicine, neurology and neurosurgery are even recognized, separate subspecialties with different training pathways. In veterinary medicine, discussions are still ongoing about the in-depth training in these subspecialties for the diplomate status. Neurologic diseases are frequently seen in veterinary practice worldwide, and increasingly better recognized and diagnosed (2). It is expected that due to the longer life expectancy of people and companion animals, neurologic problems will become more and more important (3). The increasing knowledge in veterinary neurology results in tremendous expectations from practitioners, specialists, and animals owners to develop or receive specialized care (4). This assumption is supported by a study about the cause of death in dogs in the United Kingdom: the most frequently attributed causes of death were neoplastic, musculoskeletal, and neurological disorders (5) reflecting the need for research in this discipline. This need is partially reflected by a constant increase of veterinary neurology publications (4) and the fast growing specialist colleges in Europe and US. In the last several years, diplomates of the European and American Colleges of Veterinary Neurology started multicenter studies to enhance the power of clinical research, find more evidence-based treatment methods (6), formulate consensus (7), and improve undergraduate and postgraduate training in veterinary neurology (8, 9). These efforts are still challenging and require good collaborations in the future to learn more about this specialty, about diseases of the central and peripheral nervous systems and to give specialized care to our animal patients. A journal and review method to support such efforts is one of the tools for future collaborations.

## Animal Species and Translational Aspects

Veterinary neurology and neurosurgery encompass many different species, which is a challenge for the specialized clinician. Besides the knowledge of different diseases, the anatomy and behavior of the examined species have to be considered. The most commonly examined species in small animal clinics is the dog, a species with a great potential in translational medicine (10). The most frequently observed diseases in dogs are spinal cord injury and epilepsy (11); and both diseases in dogs are considered to be valuable spontaneously occurring animal models. By contrast, cats are less frequently observed in the neurology clinic and the most frequently attributed causes of mortality in cats of all ages were trauma, renal disorders, non-specific illness, and neoplasia (12). Nevertheless, infectious or degenerative diseases of the nervous system of cats are observed in daily clinical life and valuable animal models. Besides companion animals, large animals have great importance in veterinary neurology. Horses and farm animals have diseases with great socio-economic impact. In horses, diagnostic workup of central nervous system diseases is comparable to that in small animals, whereas, in farm animals infectious diseases with zoonotic potential have to be considered, such as bovine spongiform encephalopathy and listeriosis (13, 14). Additionally, exotic animals have to be examined and treated by veterinary neurologists. It remains a challenge to enhance the knowledge of neurological diseases in all animal species.

## Advanced Diagnostic Techniques

The founder of clinical neurology in veterinary medicine was Dexler in the nineteenth century (15). At this time, knowledge in neuroanatomy and neuropathology was the basic requirements for a reliable interpretation of morbid entities of the central and peripheral nervous systems (15). This statement is still true, however, in the clinical diagnostic workup of neurological diseases several new techniques have been introduced in the last decades and are constantly being refined. The best example is the introduction of advanced imaging methods. Since magnetic resonance imaging (MRI) was introduced as a routine diagnostic method, new diseases or diseases rarely seen by neuropathologists, such as hemorrhagic and ischemic brain infarcts, have been better described (16). Animals with these disorders were surviving and recovering from the insult, which for the first time could be better defined. The challenge for the future will be to further refine such imaging methods and introduce them into daily clinical work. MRI has some powerful sequences reflecting the damage on a cellular and molecular basis as well as providing information about tissue architecture (17, 18). The same statements are consistent with new examination techniques in the field of neurophysiology. Electrodiagnostic testing is a routine method in peripheral nervous system diseases and muscular disorders. However, newer methods such as transcranial magnetic stimulation (TMS) are only now being used more often in clinical diagnostic evaluations. With this fast and non-invasive method, the functional integrity of the spinal cord can be evaluated (19). Central motor pathways from motor cortex pyramidal cells to the muscle are stimulated and the resulting magnetic motor evoked potentials (MMEPs) are measured (20, 21). More experience is still needed in veterinary medicine to apply this method for clear prognostic statements. Not only does TMS need to be further evaluated but also electroencephalography (EEG). To routinely use this powerful technique, which has been evaluated in animals for about 50 years, new protocols and electrodes have to be developed to apply it to the diagnostic workup or treatment monitoring of epilepsy and movement disorders. Several groups of researchers have started already to improve this method for diagnostic purposes (22). The challenge in the future will be to bring together all the new information, make it applicable for daily life and examine enough patients to evaluate the power of the newly modified method.

## Etiology and Pathogenesis

In veterinary neurology, several diseases still are considered “of unknown origin” or “of unknown etiology.” The best examples are some inflammatory diseases of the nervous system. Despite efforts to detect infectious agents, determine pathogenesis, and discover new aspects of neuroimmunology in inflammatory diseases, the challenge for the future still remains to find their cause and treatment. Until now treatment has been symptomatic and focuses on the suppression of an aberrant immune reaction. The immune response of dogs and humans is similar, so besides being a successful animal model for spinal cord injury and epilepsy, the dog is also considered as a useful translational model for neuroimmunological studies (23).

Some success in finding the causes of neurological diseases in animals has been achieved with genetic studies (24, 25). These publications further emphasize the value of multicenter studies in advancing knowledge in the fascinating field of veterinary neurology. Genetic analysis and DNA testing in veterinary neurology are new and powerful tools that already have elucidated several rare diseases (26). Additionally, animals with naturally occurring diseases can serve as useful animal models (27, 28) or enlighten the genetic background of fundamental blueprints, such as that which determines the number of cervical vertebrae in an animal (29). In this study, a bovine mutation model with clinical and pathological spinal cord disease revealed information about the complex processes occurring during vertebral development.

Challenges in veterinary neurology and neurosurgery in the next years will include the postgraduate education of young scientists. We face many obstacles, including funding, to keep young neurologists in a research environment. The development of veterinary neurology has such great socio-economic relevance for our animals and for the discovery of new translational models that expanding our human resources is critical to our ability to meet the challenges and opportunities in neurologic research. Stimulating specialists to work in this fascinating field to share their experience will be a major step toward improving the health of both animals and people. The new specialty “Veterinary Neurology and Neurosurgery” with the interactive review platform and the different article types will support and stimulate both clinical discussions, research in the broad field of veterinary neurology and neurosurgery and discussions about the visions of postgraduate education (see also mission statement).

## Conflict of Interest Statement

The author declares that the research was conducted in the absence of any commercial or financial relationships that could be construed as a potential conflict of interest.
